# Awareness of zoonotic diseases and parasite control practices: a survey of dog and cat owners in Qatar

**DOI:** 10.1186/s13071-018-2720-0

**Published:** 2018-03-20

**Authors:** Ana Margarida Alho, Clara Lima, Vito Colella, Luís Madeira de Carvalho, Domenico Otranto, Luís Cardoso

**Affiliations:** 1 CIISA, Faculdade de Medicina Veterinária, Universidade de Lisboa, Lisbon, Portugal; 2 Hospital Parkview Pet Center - Veterinary Clinic, Doha, Qatar; 3 Dipartimento di Medicina Veterinaria, Università degli Studi di Bari, Bari, Italy; 4 Department of Veterinary Sciences, School of Agrarian and Veterinary Sciences, University of Trás-os-Montes e Alto Douro (UTAD), Vila Real, Portugal

**Keywords:** Cats, Dogs, Parasites, Public health, Qatar, Doha, Zoonosis

## Abstract

**Background:**

Qatar is one of the wealthiest and fastest growing economies in the world, experiencing a rapid increase in human and pet populations. Given the paucity of data on prophylactic measures against endo- and ectoparasites of pets in Qatar, as well as on the owners’ awareness of zoonotic diseases, a questionnaire was conducted.

**Methods:**

From July to November 2017, 150 multiple-choice questionnaires were administered to dog and/or cat owners who attended two veterinary clinics in Doha.

**Results:**

Only 54% (81/150) owners were aware of transmittable diseases between animals and humans. “Zoonosis/zoonotic disease(s)” was unknown for 88% (132/150) of the respondents and almost a quarter had no idea of transmission pathways associated with parasitic diseases. Thirteen owners (8.7%) reported to have suffered from zoonotic diseases (10 had dermatophytosis, 2 cat-scratch disease and 1 an unknown tick-borne disease) and 24.7% had dewormed themselves. Approximately 83% had their pets yearly vaccinated and 51% identified endo- and ectoparasites on their pets. Only 10% had their animal faeces tested for intestinal parasites as requested by a veterinarian. As for internal parasite control, only 19.3% dewormed their pets with the recommended treatment regimen (minimum quarterly); 52.7% (79/150) dewormed every 4 months to 1 year; 10% (15/150) without periodicity and 8% (12/150) had never done it. For external parasite control, only 16% (24/150) treated their pets with ectoparasiticides on a monthly basis; 44.7% (67/150) every 2 months to 1 year; 6.7% (10/150) without periodicity and 24.7% (37/150) had never done it. Approximately two thirds (63.3%) of pets were allowed to sleep in the owner’s bed and 60% to lick their owner’s face. Almost all pets were fed with dry/canned food, but 4.7% were fed with raw meat. Approximately 79.5% of dog owners collect their pet’s faeces from public areas.

**Conclusions:**

These results highlight the need to raise pet owners’ awareness towards prophylactic measures to minimize the potential impact of zoonotic diseases on the health of both animals and humans in Qatar.

**Electronic supplementary material:**

The online version of this article (10.1186/s13071-018-2720-0) contains supplementary material, which is available to authorized users.

## Background

Qatar is one of the fastest and wealthiest growing economies in the world, with a rapid demographic development and a consequent increase in the animal populations. The number of dogs and cats kept as pets has also increased, as well as the number of animals that have been imported and exported from all over the world [1, 2].

Changing demographics and concomitant human behaviour tend to favour the emergence and spread of zoonoses [3]. In the modern-day society, the human-animal bond has become stronger with pets playing an important role as a source of companionship, entertainment and emotional support to their owners. Nevertheless, this close contact may also increase the risk of exposure to infectious diseases, as pets have been implicated in the transmission of more than 60 zoonotic agents [4]. To overcome such potential hazards, owners must be informed about risk factors from such a close relationship, and educated about strategies to protect themselves and their animals. Known risk factors for infection include lack of regular and efficient application of endo- and ectoparasiticides, absence of routine vaccination programs, poor hygiene practices, low socio-economic factors and education, high animal density, improper cooking of food, geophagia (especially in children), failure to regularly pick up and dispose faeces, lack of dog and cat population control measures and consequent high numbers of free-ranging dog and cat populations [4].

Little information is currently available regarding prophylactic measures against parasites and vaccination programs in pets in Qatar. Similarly, limited data are accessible on the degree of pet owners’ awareness regarding zoonotic diseases. Therefore, a questionnaire was conducted to several clients in veterinary clinics of Doha, the capital and most populated city of Qatar.

## Methods

### Animals and samples

From July to November 2017, a multiple-choice questionnaire (Additional file 1: Figure S1) written in English was administered to pet (dog and/or cat) owners (*n* = 150) who attended two veterinary medical centres located in the residential centre of Doha. Thirty questionnaires had been pre-tested to assess the suitability of different survey formats and questions (written and multiple-choice answers). The final format was a multiple-choice based interview that took approximately 6–10 min to complete (Additional file 1: Figure S1).

Information on the owners (i.e. gender, nationality, residence, profession and a previous zoonotic disease) was collected, as well as on the animal species (dog/cat), age, breed, number of animals in the household, pet’s origin (shelters or pet shops/“souks”/markets or imported), feeding habits, pet’s indoor/outdoor activity, number of visits to the veterinarian and reasons for appointments, frequency of vaccination and regularity of endo- and ectoparasites prevention. Other questions included the owner’s knowledge regarding potential zoonotic diseases, i.e. if they had ever suffered from a zoonotic disease; if they were aware of the terms “zoonosis/zoonotic disease(s)”; if they had ever been treated against intestinal parasites; and their perception of potential pathways/vehicles associated with diseases transmission between people and pets.

Deworming schedules and protocol guidelines from the European Scientific Counsel Companion Animal Parasites (ESCCAP) and from the Tropical Council for Companion Animal Parasites (TroCCAP) were considered to determine the most appropriate number of ecto- and endoparasiticide treatment administrations: at least quarterly for worm control (without faecal analysis) and monthly for ectoparasite control [5–8].

### Statistical analysis

The Chi-square test and Fisher’s exact test (FET) were used to compare proportions, with a *P*-value < 0.05 regarded as statistically significant. Analyses were performed with SPSS®21 for Windows.

## Results

### Study population characterisation

Overall, out of the total respondents, 51.3% (77/150) were sole cat owners, 30.7% (46/150) sole dog owners and 18% (27/150) were both cat and dog owners. Concerning the nationality of interviewees, they came from 31 origin countries, with British as the most representative nationality with 24% (36/150), followed by 16% (24/150) Qataris and 8% (12/150) Indians. The average age of the respondents was 37.5 years old (standard deviation: 10.9), with females representing 62.7% (94/150) of the owners questioned.

The most popular pet in Qatar was found to be the cat, with 69.3% (104/150) of the respondents having at least one cat and 51.3% of owners having only cats as their companion animals. Cat households had an average of 2.4 cats, ranging from 1 to 11. Out of the 251 cats of this study, the Domestic Shorthair was the most popular breed (166/251), followed by Persian (34/251). Breeds like Scottish Fold, British Shorthair and Longhair, Turkish Angora, Himalayan and Sphynx were also reported.

Dog owners represented 48.7% of the respondents, with 73/150 as having at least one dog. Around 30.1% (46/150) of the households had only dogs as a companion animal. Dog households had an average of 1.8 dogs, ranging from 1 to 7. Out of the 127 dogs of this study, Saluki and mixed-Saluki represented the majority of the dog breeds (31/127), followed by unspecific breed (17/127), Labrador Retriever (9/127), Golden Retriever (7/127). Cocker Spaniel, German Shepherd, Pomeranian, Dachshund, Schnauzer and Havanese were also reported. Additionally, 18% (27/150) of the households had both cats and dogs as companion animals.

### Origin of dogs and cats

Rescued animals represented 44% (66/150) of the population, as the majority of the respondents had either adopted their pets from the streets [32.7% (49/150)] or from shelters [11.3% (17/150)] in Qatar. In addition, 24% (36/150) of the owners imported their pets from foreign countries to Qatar (i.e. Australia, Bahrain, Brazil, Denmark, Dubai, Egypt, France, Hungary, India, Malaysia, Poland, Portugal, the UK, Ukraine and the USA), with the UK and Ukraine as the most common sources for these pets (22.2% and 19.4%, respectively). Pets purchased from shops [6.7% (10/150)], markets (“souks”) [7.3% (11/150)] and breeders [1.3% (2/150)] represented 15.3% (23/150) of the pet population.

### Reasons for veterinary appointment

The major reason why owners visit the veterinarian was for vaccinations [82% (123/150)], followed by pet sickness [38.7% (58/150)], regular health checks [24% (36/150)], pet check/ blood tests and necessary prophylaxis (vaccination/deworming) prior travel [16% (24/150)], deworming administration [13.3% (20/150)] and grooming [6% (9/150)] (Table 1).

**Table 1:** Reasons for veterinary appointment

Reason	*n*	%	*P*-value
Vaccine	123	82.0	
Illness	58	38.7*	*P* < 0.0001^a^
Health check	36	24.0*	*P* = 0.006^b^
Travel	24	16.0	
Deworming	20	13.3	
Grooming	9	6.0*	*P* = 0.032^c^

*Statistically significant difference to the frequency value positioned immediately above

^a^*χ*^2^ = 7.67, *df* = 1

^b^*χ*^2^ = 2.74, *df* = 1

^c^*χ*^2^ = 2.15, *df* = 1

### Vaccination and deworming practices

Approximately 83% of the respondents (124/150) had their pets on a yearly vaccination program, with 64.7% believing that the vaccination of pets protects both human and animal health, 18.7% that it protects only animals and 2% for their own protection (Table 2).

**Table 2:** Goals of pet vaccination

Goal	*n*	%	*P*-value
Protect animal and human health	97	64.7	
Protect only animals	28	18.7*	*P* < 0.0001^a^
Protect only humans	3	2.0*	*P* < 0.0001^b^
na	23	15.3	Not computed

*Statistically significant difference to the frequency value positioned immediately above

^a^*χ*^2^ = 8.10, *df* = 1

^b^Fisher’s exact test

*Abbreviation*: *na* no answer

Regarding internal parasite control, deworming practices have been put in practice with the recommended treatment regimen (minimum quarterly) by only 19.3% (29/150) of the owners, 52.7% (79/150) dewormed every 4 months to 1 year, 10% (15/150) with no defined pattern of frequency and 8% (12/150) had never internally dewormed their pets (Table 3). For ectoparasites prevention, only 16% (24/150) treated their pets with ectoparasiticides on a monthly basis, 44.7% (67/150) every 2 months to 1 year, 6.7% (10/150) with no defined pattern of frequency and 24.7% (37/150) had never used external parasite preventatives (Table 3). Nevertheless, 51% of the respondents claimed to have identified endo- and ectoparasites on their pets: 12% have seen fleas, 11.3% ticks and 8% intestinal worms. In addition, only 10% (15/150) of the pet owners have been asked by the assisting veterinarian for a faecal analysis of their pets to check for intestinal parasites. However, 24.7% (37/150) of the owners claimed to have dewormed themselves, half of them by their own accord and the remaining under doctor’s prescription.

**Table 3:** Frequency of internal and external parasite control

Frequency	*n*	%	*P*-value
Internal parasites			
Every 1–6 times a year^e^	79	52.7	
Every month	29	19.3*	*P* < 0.0001^a^
No defined pattern of frequency^f^	15	8.0*	*P* = 0.022^b^
Never	12	10.0	
na	15	8.0	Not computed
External parasites			
Every 1–6 times a year^e^	67	44.7	
Never	37	24.7*	*P* = 0.0003^c^
Every month	24	16.0	
No defined pattern of frequency^f^	10	6.7	*P* = 0.011^d^
na	12	8.0	Not computed

*Abbreviation*: *na* no answer

*Statistically significant difference to the frequency value positioned immediately above

^a^*χ*^2^ = 6.01, *df* = 1

^b^*χ*^2^ = 2.28, *df* = 1

^c^*χ*^2^ = 3.64, *df* = 1

^d^*χ*^2^ = 2.55, *df* = 1

^e^Every 2 months, every 3 months, every 4 months, every 6 months or every 1 year

^f^Whenever necessary/as remembered/first time

### Knowledge on zoonoses

Of the 150 owners surveyed, 54% (81/150) were aware of transmittable diseases between animals and humans (Table 4). When questioned about the meaning of the term “zoonosis/zoonotic disease(s)”, 88% of the owners (132/150) had never heard of it before. Of the 12% (18/150) that had, only 15 were aware of its meaning and able to describe it correctly. No further statistical association was found between the category profession and the meaning of “zoonosis/zoonotic disease(s)”. Dermatophytosis (ringworm) [21.3% (32/150)], rabies [16% (24/150)] and toxoplasmosis [7.3% (11/150)] were the most cited examples (Table 5). People with their residence in the outskirts were more aware of dermatophytosis than those living in Doha (44.4 *vs* 18.5%, respectively; FET: *P* = 0.027). In addition, people who reported to have previously suffered from a zoonotic disease were also more aware of dermatophytosis than those not affected by a zoonotic disease (69.2 *vs* 16.8%, respectively; FET: *P* < 0.0001).

**Table 4:** Awareness of diseases transmissible between animals and people, and knowledge of zoonosis/zoonotic disease(s) among 150 individuals according to their gender, nationality, residence, profession and previous affection by a zoonotic disease

Variable/category	Percentage (no.) of positive responses	
	Aware of diseases transmissible between animals and people	Heard of zoonosis/zoonotic disease(s)
Gender		
Female	57.4 (54)	12.8 (12)
Male	48.2 (27)	10.7 (6)
Nationality		
Qatari	37.5 (9)	4.2 (1)
Other	57.1 (72)	13.5 (17)
Residence^a^		
Doha	50.0 (65)	10.8 (14)
Outside	78.8 (14)	22.2 (4)
Profession		*P* = 0.027^a^
Health-related	81.2 (13)	31.2 (5)
Other	50.7 (68)	9.7 (13)
Previous zoonotic disease	*P* = 0.016^b^	
Yes	92.3 (12)	15.4 (2)
No	50.4 (69)	11.7 (16)
Total	54.0 (81)	12.0 (18)

^a^Fisher’s exact test (accounted only for 148 individuals)

^b^*χ*^2^ = 5.77, *df* = 1. Only statistically significant differences are shown

**Table 5:** Awareness of zoonotic diseases (i.e. whose agent is transmissible between animals and people)

Disease	*n*	%	*P*-value
Dermatophytosis	32	21.3	
Rabies	24	16.0	
Toxoplasmosis	11	7.3*	*P* = 0.019^a^
Worms	9	6.0	
Bartonellosis	3	2.0	
Lyme disease	3	2.0	
Bird flu	2	1.3	
Diarrhea	2	1.3	
Ebola	1	0.7	
Leptopirosis	1	0.7	
Lice	1	0.7	
Ticks	1	0.7	

*Statistically significant difference to the frequency value positioned immediately above

^a^*χ*^2^ = 2.34, *df* = 1

Veterinary practitioners (*n* = 6), magazines/books (*n* = 6), internet (*n* = 4) and school (*n* = 4) were the most common sources of information referred. Additionally, of those 10.7% (16/150) who worked in the healthcare field (e.g. dentists, nurses, physicians, paramedics and veterinarians), only 12 were able to give examples of diseases transmittable between animals and humans, only five had heard of “zoonosis/zoonotic disease(s)” and just four were able to define its meaning. Thirteen owners (8.7%) had suffered from zoonotic diseases, of which 10 from dermatophytosis, two from cat-scratch disease and one from an unknown tick-borne disease. In three out of these 10 infections, owners had a health profession-related illness (i.e. one dermatophytosis, one other cat-scratch disease and the other an unknown tick-borne disease).

When questioned about possible pathways of transmission of parasites to animals, 73 indicated animal faeces, 57 raw meat, 40 soil samples, 39 food items, 37 arthropods, 31 mother-to-child, 20 plants, 18 environmental contamination and one claw scratch. Almost a quarter of the respondents [24.7% (37/150)] had no idea of pathways of transmission (Table 6). Health care providers indicated animal faeces (75.0 *vs* 45.5%; *χ*^2^ = 3.86, *df* = 1, *P* = 0.049), raw meat (75.0 *vs* 33.6%; *χ*^2^ = 8.72, *df* = 1, *P* = 0.003), soil (68.8 *vs* 21.6%; FET: *P* < 0.0001) and plants (31.2 *vs* 11.2%; FET: *P* = 0.042) more frequently than people with non-health-related professions.

**Table 6:** Indication of possible pathways associated with parasitic diseases transmitted to animals

Pathway/substance	*n*	%	*P*-value
Animal faeces	73	48.7	
Raw meat	57	38.0	
Soil	40	26.7*	*P* = 0.036^a^
Food	39	26.0	
Arthropods	37	24.7	
No idea	37	24.7	
Mother to child	31	20.7	
Plants	20	13.3	
Environmental contamination	18	12	
Claw scratch	1	0.7*	*P* < 0.0001^b^

*Statistically significant difference to the frequency value positioned immediately above

^a^*χ*^2^ = 2.10, *df* = 1

^b^Fisher’s exact test

### Pet management

Although almost all dogs and cats were fed with dry and/or canned pet food, still 4.7% (7/150) of the owners fed their pets with raw meat. 73.3% (110/150) of the household pets were allowed to have access to their owners’ bedroom, 63.3% (95/150) to sleep in the owner’s bed and 60% (90/150) to lick the owner’s face. Of the total respondents, 61.3% (92/150) keep their animals strictly indoors, 35.3% (53/150) indoors with outdoor access and 3.3% (5/150) have them exclusively outdoors (Table 7). Around 95.8% of the owners walk their dogs outside the house in public spaces, i.e. streets, parks and beaches, 46.6% either on or off leash (34/73), 43.8% (32/73) exclusively on leash and 6.8% (5/73) exclusively off leash. Approximately 79.5% (58/73) of dog owners claimed to collect their pet faeces in public areas. Regarding the remaining ones, 5.5% state that they collect it only when it occurs on pathways, 4.1% only when observed, 2.7% only when they carry a bag and 2.7% never. Regarding the maintenance frequency of the cat litter trays, 63.8% claimed to clean it daily, 14.9% every 2 days, 14.9% every 3 days and 6.4% every 3 days or more.

**Table 7:** Habitat of pets according to the information provided by their owners

Habitat	*n*	%	*P*-value
Indoors	92	61.3	
Indoors and outdoors	53	35.3*	*P* < 0.0001^a^
Outdoors	5	3.3*	*P* < 0.0001^b^

*Statistically significant difference to the frequency value positioned immediately above

^a^*χ*^2^ = 4.51, *df* = 1

^b^Fisher’s exact test

## Discussion

Results of this study demonstrated that in Qatar pet owners have a low awareness of zoonotic diseases and parasite control practices of dogs and cats. Although most of the owners administered antiparasitic drugs to their pets, results show that this occurs at irregular intervals, which may render them ineffective. The studied population showed concerns towards pets’ vaccination with 83% of the owners having their pets annually vaccinated. However, only 16% treated their animals monthly against external parasites and almost one quarter had never done it. These results contrast with other international studies, namely from Portugal [9], where 92.2% of the dogs surveyed were treated against external parasites [although all-year round or seasonally (at monthly intervals) in just 50.5%] and 63.6% of the cats were treated with ectoparasiticides (although the majority at non-frequent intervals). The obtained results are worrying for canine and feline health, considering that the occurrence of *Anaplasma platys*, *Babesia gibsoni*, *Babesia vogeli*, *Ehrlichia canis*, *Hepatozoon canis* and *Mycoplasma* spp. has been reported in domestic dogs, and *Babesia felis*, *B. vogeli*, “*Candidatus* Mycoplasma haemominutum”, *E. canis* and *Mycoplasma haemofelis* in domestic cats from Qatar [10].

Internal parasite treatment and prevention was irregularly performed, with only 19.3% of the pet owners deworming their pets at the recommended frequency (quarterly) and more than a half (52.7%) deworming every 4 months to 1 year. These results contrast with other international studies, namely from Australia, where a much higher anthelmintic administration frequency was found, with 54% of dogs being dewormed quarterly [11]. The low percentage of pets under effective parasitic prophylaxis is worrying considering the results observed by Abu-Madi et al. [12, 13], who found a high prevalence of intestinal helminths in stray cats from Doha and its outskirts. Although data obtained from stray cats cannot be extrapolated to client-owned cats, the parasitic diversity and burden previously detected in Doha [12, 13] is relevant considering the high number of pets that have been rescued from the streets and shelters in the country. The low frequency by which anthelmintics are being given (once, twice or thrice a year) does not guarantee an adequate protection of the pet against these parasitic diseases [14, 15]. Only for 10% of the cases, the veterinarian had asked for pet’s faecal analyses. It is crucial that local veterinarians and researchers encourage faecal analysis and that further investigation is undertaken to have a better knowledge on the prevalence of intestinal parasites in pets in Qatar.

Despite the potential occurrence of zoonotic parasites previously detected in the country [10, 12, 13], the majority of the pet owners were not aware of zoonotic diseases. Although numerous respondents were native English speakers, Arabic speakers may have possibly misunderstood some issues on questions, which might have influenced the final results. Further questionnaires should include a version in the Arabic language to allow for full comprehension by non-English speaking people.

**Fig. 1:**
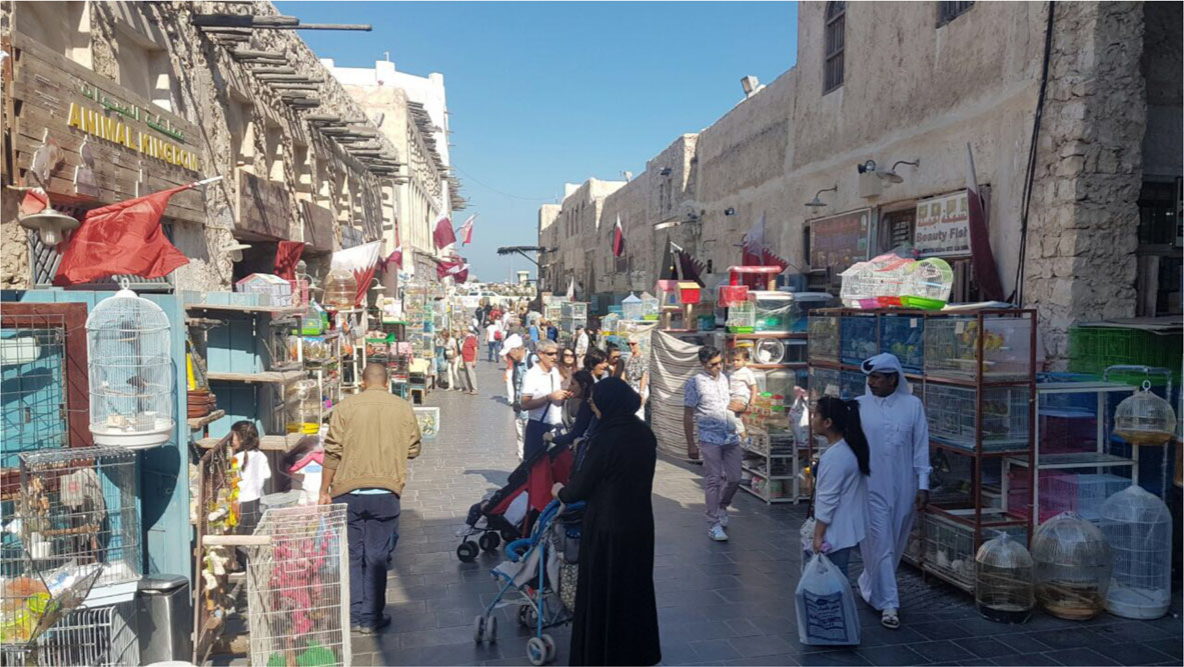
The main animal “souk” market of Doha, Souk Waqif

Veterinarians should play a central role in the promotion of pet owners’ education towards zoonotic diseases and about proper treatment and prevention strategies [16]. Additionally, to prevent zoonotic disease transmission and assure healthy adoption and sale of pets, tight regulation towards animal adoption, exchange, purchase and importation should be implemented in local pet shops and animal “souk” markets (Fig. 1) to reduce the occurrence of dermatophytosis and intestinal parasites. Basic prophylaxis such as deworming, vaccination and proper environmental hygiene should be performed prior to the adoption of animals from shelters and pet shops. Continuous stray and feral dog and cat population control programs are also essential to decrease the transmission and perpetuation of infectious diseases [9, 17, 18].

Considering that the number of imported pets from foreign countries to Qatar represents at least 22% of the population in this study, treatment of all imported animals against intestinal helminths and ectoparasites upon arrival is critical to prevent introduction of non-endemic parasites. The same recommendation should be implemented on pets relocating from or travelling to foreign countries (only 16.7% of the respondents claim to take their animals for health checks before travelling). Aside from intestinal helminths and protozoans in the stray animal population in Qatar, a screening for vector-borne diseases should be implemented as part of the routine health check of all animals being exported from Qatar, especially when they are being relocated in countries where these diseases are not endemic [18].

Additionally, some behavioural practices reported, such as feeding their pets with raw meat or the close physical contact between owners and pets, show a lack of knowledge regarding animal and public health issues. In the households assessed in Doha, 73.3% of the pets were allowed to have access to their owners’ bedroom, 63.3% to sleep in the owner’s bed and in 60% to lick the owner’s face. These results are similar to those found by Matos et al. [19], who reported that in Portugal dogs were allowed to visit the owners’ bedroom in 82.4% of the households, to sleep with the owners in their beds in 43.1% of the households and to lick the owner’s face in 75.5% of the cases. It is important to highlight that these habits increase the risk for the transmission of zoonotic diseases.

Regarding the collection of dog faeces, almost 79.5% of the owners claimed to collect them. This means that approximately one fifth (20.5%) of the owners were not performing it, which is considerably lower than the 37% reported in Portugal [9] and the 39% reported in the Netherlands [20]. The percentage found may be overestimated (i.e, not reflecting owners’ real behaviour), as this is a sensitive matter. Nonetheless, this measure should be encouraged, as it is an extremely relevant and easy way to reduce environmental contamination in order to safeguard public and animal health.

## Conclusions

This study identified several risk factors for the transmission of parasitic zoonoses associated with pet ownership in Qatar. Other relevant risk factors such as the presence of children or immunocompromised members in the family, slaughter practices, drinking water sources and education level (basic, intermediate, academic) should also be included in future analyses. It might be useful to extend this study to owners living in rural environments and compare the results, practices and risk factors with those living in the city center. Our results highlight the need to raise pet owners’ awareness toward transmittable diseases and effective prophylactic measures to minimize the risk of zoonotic diseases in Qatar.

## Additional file

Additional file 1: **Figure S1.** Multiple-choice questionnaire administered to dog and/or cat owners who attended the veterinary medical centres surveyed in the residential centre of Doha. (DOCX 121 kb)

## Abbreviations

ESCCAP: European Scientific Counsel Companion Animal Parasites
TroCCAP: Tropical Council for Companion Animal Parasites
FET: Fisher’s exact test

## References

[1] Abu-Madi MA, Behnke JM, Boughattas S, Al-Thani A, Doiphode SH, Deshmukh A (2016). Helminth infections among long-term-residents and settled immigrants in Qatar in the decade from 2005 to 2014: temporal trends and varying prevalence among subjects from different regional origins. Parasit Vectors.

[2] Ministry of Development Planning and Statistics. Qatar in figures 2017 (32nd issue). In: Publications, knowledge Centre, MPDS; 2017. https://www.mdps.gov.qa/en/pages/default.aspx. Accessed 12 Nov 2017.

[3] Abu-Madi MA, Behnke JM, Ismail A, Boughattas S (2016). Assessing the burden of intestinal parasites affecting newly arrived immigrants in Qatar. Parasit Vectors.

[4] Macpherson CN (2005). Human behaviour and the epidemiology of parasitic zoonoses. Int J Parasitol.

[5] European Scientific Counsel Companion Animal Parasites (ESCCAP). Guideline 01 - worm control in dogs and cats (3rd ed.). 2017. http://www.esccap.org/uploads/docs/0x0o7jda_ESCCAP_Guideline_01_Third_Edition_July_2017.pdf. Accessed 5 Nov 2017.

[6] European Scientific Counsel Companion Animal Parasites (ESCCAP). Guideline 03 - control of ectoparasites in dogs and cats (5th ed.). 2016. http://www.esccap.org/uploads/docs/uswsanew_ESCCAP_Guideline_03_Fifth_Edition__April_2016.pdf. Accessed 5 Nov 2017.

[7] European Scientific Counsel Companion Animal Parasites (ESCCAP). Guideline 05 - control of vector-borne diseases in dogs and cats (2nd ed.). 2012. http://www.esccap.org/uploads/docs/ih38c2d6_ESCCAP_Guidelines_GL5_01Oct2012.pdf. Accessed 5 Nov 2017.

[8] Tropical Council for Companion Animal Parasites (TroCCAP). Guidelines for the diagnosis, treatment and control of canine endoparasites in the tropics (1st ed.). 2017. http://www.troccap.com/2017press/wp-content/uploads/2017/10/TroCCAP_canine_endo_guidelines_Ver1.pdf. Accessed 8 Nov 2017.

[9] Matos M, Alho AM, Owen SP, Nunes T, Madeira de Carvalho L (2015). Parasite control practices and public perception of parasitic diseases: a survey of dog and cat owners. Prev Vet Med.

[10] Alho AM, Lima C, Latrofa MS, Colella V, Ravagnan S, Capelli G (2017). Molecular detection of vector-borne pathogens in dogs and cats from Qatar. Parasit Vectors.

[11] Palmer CS, Robertson ID, Traub RJ, Rees R, Thompson RC (2010). Intestinal parasites of dogs and cats in Australia: the veterinarian's perspective and pet owner awareness. Vet J.

[12] Abu-Madi MA, Al-Ahbabi DA, Al-Mashhadani MM, Al-Ibrahim R, Pal P, Lewis JW (2007). Patterns of parasitic infections in faecal samples from stray cat populations in Qatar. J Helminthol.

[13] Abu-Madi MA, Pal P, Al-Thani A, Lewis JW (2008). Descriptive epidemiology of intestinal helminth parasites from stray cat populations in Qatar. J Helminthol.

[14] Kopp SR, Coleman GT, Traub RJ, McCarthy JS, Kotze AC (2009). Acetylcholine receptor subunit genes from *Ancylostoma caninum*: altered transcription patterns associated with pyrantel resistance. Int J Parasitol.

[15] Bowman DD (2012). Heartworms, macrocyclic lactones, and the specter of resistance to prevention in the United States. Parasit Vectors.

[16] Day MJ (2011). One health: the importance of companion animal vector-borne diseases. Parasit Vectors.

[17] Pereira A, Martins Â, Brancal H, Vilhena H, Silva P, Pimenta P (2016). Parasitic zoonoses associated with dogs and cats: a survey of Portuguese pet owners' awareness and deworming practices. Parasit Vectors.

[18] Otranto D, Dantas-Torres F, Mihalca AD, Traub RJ, Lappin M, Baneth G (2017). Zoonotic parasites of sheltered and stray dogs in the era of the global economic and political crisis. Trends Parasitol.

[19] Ferreira A, Alho AM, Otero D, Gomes L, Nijsse R, Overgaauw PAM (2017). Urban dog parks as sources of canine parasites: contamination rates and pet owner behaviours in Lisbon, Portugal. J Environ Public Health.

[20] Overgaauw PA, van Zutphen L, Hoek D, Yaya FO, Roelfsema J, Pinelli E (2009). Zoonotic parasites in fecal samples and fur from dogs and cats in The Netherlands. Vet Parasitol.

