# Supplementary material for: Awareness of zoonotic diseases and parasite control practices: a survey of dog and cat owners in Qatar
Source: Parasit Vectors. 2018 Mar 20;11:133. doi: 10.1186/s13071-018-2720-0 (PMC5859551; doi:10.1186/s13071-018-2720-0)
Supplement: Supplementary file 1 — Figure S1. Multiple-choice questionnaire administered to dog and/or cat owners who attended the veterinary medical centres surveyed in the residential centre of Doha. (DOCX 121 kb) [file 13071_2018_2720_MOESM1_ESM.docx]

THIS IS AN ANONYMOUS SURVEY REGARDING GENERAL PUBLIC KNOWLEDGE ON PETS HEALTH. THE RESULTS OF THIS SURVEY WILL BE USED FOR DATA ANALYSIS AND FUTURE EDUCATIONAL PROGRAMS ON RESPONSIBLE PET OWNERSHIP.

THE QUESTIONNAIRE WILL TAKE ONLY 5 MINUTES TO BE COMPLETED. THANK YOU FOR YOUR COLLABORATION!

| What is your Nationality? _________  What is your Profession? _________  How old are you? _______________ | What is your Gender? M / F  Where do you live in Qatar? _________  Date: ___________________________ |
| --- | --- |
| **I HAVE DOGS** | **I HAVE CATS** |
| - HOW MANY DOGS DO YOU HAVE? _________ | - HOW MANY CATS DO YOU HAVE? _________ |
| - WHERE DID YOU GET YOUR DOG(S) FROM?   Rescue - Streets - Pet Shop - Souk - Friends - Imported from: _________ | - WHERE DID YOU GET YOUR CAT(S) FROM?   Rescue - Streets - Pet Shop - Souk - Friends - Imported from: _________ |
| - WHAT IS/ ARE YOUR DOG(S) GENDER? _________   Intact male – Neutered male – Intact female – Neutered female | - WHAT IS/ ARE YOUR CAT(S) GENDER?   Intact male – Neutered male – Intact female – Neutered female |
| - WHAT IS/ ARE YOUR DOG(S) BREED? _________ | - WHAT IS/ ARE YOUR CAT(S) BREED? _________ |
| - HOW OLD IS/ ARE YOUR DOG(S)? _________ | - HOW OLD IS/ ARE YOUR CAT(S)? _________ |
| - WHAT DO YOU FEED YOUR DOGS WITH?   Canned food for Dogs - Dry Food for Dogs - Homemade cooked food - Raw meat | - WHAT DO YOU FEED YOUR CATS WITH?   Canned food for Cats - Dry Food for Cats - Homemade cooked food - Raw meat |
| - HOW MANY TIMES IN A YEAR DO YOU TAKE YOUR DOG(S) TO THE VET?   Every month - Every 3 months - Every 6 months - Once a year - Other: _________ | - HOW MANY TIMES IN A YEAR DO YOU TAKE YOUR CAT(S) TO THE VET?   Every month - Every 3 months - Every 6 months - Once a year - Other: _________ |
| - WHAT ARE THE REASONS WHY YOU TAKE YOUR DOG(S) TO THE VET?   Vaccines - Travel - Chronic Illness - Regular Check Up - Other: _________ | - WHAT ARE THE REASONS WHY YOU TAKE YOUR CAT(S) TO THE VET?   Vaccines - Travel - Chronic Illness - Regular Check Up - Other: _________ |
| - HOW OFTEN DO YOU VACCINATE YOU DOG(S)?   Every year - When I remember – Other: _________ | - HOW OFTEN DO YOU VACCINATE YOU CAT(S)?   Every year - When I remember - Other: _________ |
| - HOW OFTEN DO YOU DEWORM YOUR DOG(S)?   Once a month - Every 4 months - Every 6 months - Every year - Other: _________ | - HOW OFTEN DO YOU DEWORM YOUR CAT(S)?   Once a month - Every 4 months - Every 6 months - Every year - Other: _________ |
| - HOW OFTEN DO YOU TREAT YOUR DOG(S) FOR FLEAS/ TICKS?   Once a month - Every 4 months - Every 6 months - Every year - Other: _________ | - HOW OFTEN DO YOU TREAT YOUR CAT(S) FOR FLEAS/ TICKS?   Once a month - Every 4 months - Every 6 months - Every year - Other: _________ |
| - HAVE YOU SEEN FLEAS OR TICKS IN YOUR DOG(S)?   Yes - No | - HAVE YOU SEEN FLEAS OR TICKS IN YOUR CAT(S)?   Yes - No |
